# IL22 drives cutaneous melanoma cell proliferation, migration and invasion through activation of miR-181/STAT3/AKT axis

**DOI:** 10.7150/jca.40974

**Published:** 2020-02-19

**Authors:** Yuanmin He, Yan Yang, Jixiang Xu, Yongmei Liao, Li Liu, Li Deng, Xia Xiong

**Affiliations:** 1 Department of Dermatology, the Affiliated Hospital of Southwest Medical University, Luzhou, Sichuan 646000, China; 2 Department of Public Health, Southwest Medical University, Luzhou, Sichuan 646000, China

**Keywords:** cutaneous melanoma, IL22, miR-181, STAT3, AKT

## Abstract

Cutaneous melanoma (CM) is neoplastic growth of melanocytes with strong potential to proliferate and invade, prone to a fatal disease soon which is beyond surgical clearance. The use of regulator involving in antitumor immune responses has been identified as a potential therapeutic option for CM, but still need fully understood at present. Recently, interleukin 22 (IL22), an immune molecule secreted mostly by CD4^+^ T cells, was reported having functions in a variety of human diseases including encouragement of lung cancer progression, yet, its role in CM is lacking. Here, we first found elevated expression of IL22 in both serum of CM patients and tissues. Up-regulated IL22 significantly promoted cell proliferation, migration and invasion in CM cells deriving from different original culture history. Moreover, *in vivo* CM model, IL22 treatment caused a significant increase in tumor size. Additionally, we found these effects accompanied by obvious increased miR-181 expression in CM. Importantly, both *in vivo* and *in vitro* results revealed that miR-181 downregulation reversed the effects of IL22 on CM cell proliferation, migration, invasion, and CM tumor size as well. Finally, in CM cells deriving from different culture history, we identified *STAT3* to be a target gene of miR-181. Higher expression level of IL22 suppressed STAT3 expression, while enhanced expression of p-AKT, p-β-catenin and MMP4; however, down-regulation of miR-181 reversed these situations. Thus, we conclude that IL22 promotes CM progression by driving miR-181/STAT3/AKT axis.

## Introduction

Cutaneous melanoma (CM) is one of the most common malignancies with high aggressive potential^1^, and it is estimated that there will be 96,480 new cases of CM and approximately 7,230 deaths from this disease in 2019 in the United States (https://seer.cancer.gov/statfacts/html/melan.html). In the last decade, despite evolving progress have been made in identifying new therapeutic options to target cell proliferation and invasion pathways^2^, the potential use of regulator involving in antitumor immune responses and the molecular basis underlying their tumorsuppressed activity are extensively being investigated for providing effective therapeutic approaches for advanced CM^3, 4^. Since the 1990s, interferon alpha and interleukin (IL)2 have been tested widespread in clinical trials, and increasing numbers of immunotherapeutic approaches have being identified as potential effective options in advanced CM treatment^5^. However, currently, there is no proven treatment with both high response and low resistance^6^. Thus, the exact immune modulator implicated in the antitumor immune responses on CM aggressiveness need more in-depth studies.

IL22, a immunoregulatory cytokine, is selectively produced by CD4^+^ T cell subsets such as Th17 cells and Th22 cells, innate lymphoid cells, natural killer T cells, γδ T cells, and rarely CD8^+^ T cells^7^, ^8^. IL22 is a distinctive member of IL10 family of cytokines, and seems to act exclusively on epithelial cells with expressing a transmembrane receptor of IL22R1 and IL10R2^9^, ^10^. The signaling pathway downstream of IL22 has not yet been fully elucidated. STAT3 cascade, inducing phosphorylation in several kinases, was considered to be a mediator of IL22-induced effects in some studies^11^, ^12^. Individual miRNAs involving in modulating CD4^+^ T cell responses also have been found critical for activation of IL22 and STAT3 signaling^13^, ^14^.

Previous studies have shown that IL-22 plays critical roles through its receptor in promotion of antimicrobial immunity, tissue repair and inflammation^15^. Recently, IL-22 has been closely related to the initiation and progression of cancer^16^. Remarkably, IL-22 signaling was identified as a critical regulator in cell proliferation, migration and apoptosis, that could be hijacked by aggressive cancers to enhance tumor growth and metastasis^12^, ^17^. However, the potential role of IL-22 in CM and underlying mechanism involved in it remain unclear.

In the present study, we noticed that IL-22 was up-regulated in both serum of CM patients and CM tissues, accompanying by significantly increased miR-181 expression. Moreover, we confirmed that up-regulated IL22 significantly promoted cell proliferation, migration and invasion in CM cells deriving from different original culture history, as well as caused a significant increase in tumor size *in vivo* CM model; however, miR-181 downregulation reversed the effects of IL22 in both vivo and vitro models. Finally, we identified *STAT3* to be a target gene of miR-181, which involving in enhanced expression of p-AKT, p-β-catenin and MMP4.

## Methods and Materials

### Patients and samples

CM tissues, adjacent normal tissues and peripheral blood were collected from 30 patients diagnosed with CM according to clinical and histological manifestation in department of dermatology, the Affiliated Hospital of Southwest Medical University (Based on the expression of interleukin 22 in normal human tissues and other tumor tissues obtained from published report^18^, ^19^, we statistical estimated sample size in our study, and found that 30 cases enrolled in both patient group and health group could meet the statistical requirement.). They are 40-60 years old with 17 male and 13 female. 13 of them were diagnosed as stage I-II CM and 17 of them were diagnosed as stage III-IV CM. Control serum were obtained from corresponding 30 healthy controls with matched age and sex from the Affiliated Hospital of Southwest Medical University. Each sample was snap-frozen using liquid nitrogen, followed by storing at a temperature of -80°C until processed. The investigational protocol was approved by the Ethics Committee of the Affiliated Hospital of Southwest Medical University according to the Declaration of Helsinki Principles.

### Cell culture and transfection

CM cell lines (A375 and M14) were obtained from Chinese Academy of Science and cultured in RPMI1640 (Gibco, Grand Island, NY, USA) medium containing 10% FBS (Gibco, Grand Island, NY, USA) with 100U/mL penicillin-streptomycin (Invitrogen, Carlsbad, CA, USA). Cells were cultured at 37°C in 5% CO2. IL22 were obtained from Sigma Chemical Co (St. Louis, MO, USA). Sh-miR-181 and control oligonucleotide were obtained from Genepharma (Shanghai, China). The oligonucleotides were transfected into A375 and M14 cells (200 nmol per well) using Lipo3000 (Invitrogen, Carlsbad, CA, USA).

### 3-(4,5-dimethylthiazol-2-yl)-2, 5-diphenyltetrazolium bromide (MTT) assay

A total of 3000 cells/well were seeded into 96-well plates and cultured for 12 h followed by transfection with sh-miR-181 or control oligonucleotide. After 24 h of transfection, IL22 (Sigma, St. Louis, MO, USA) was added into each well and cultured for 24 h, MTT assay was used to assess the cell viability. 0.5% MTT (Sigma, USA) was added to the culture medium at 37°C for 4 h. The supernatant was removed and DMSO (Gibco, CA, USA) was added into each well. After that, the absorption at 490 nm was evaluated using microplate reader (BioRad, CA, USA).

### ELISA

Human IL22 Quantikine ELISA kits was used to analyze patient's serum samples, following the manufacturer's instructions (R&D Systems).

### Clonogenic assay

The cells treated with indicated conditions and then seeded in 12-well plates (100/well). After incubated for 2 weeks, crystal violet (0.05%, Beyotime, Shanghai, China) was used to stain the colonies. Colonies with sizes ≥ 1 mm were counted.

### Wound healing

The cells treated with indicated conditions and then seeded in 6-well plates (1×10^4^/well). After cultured in serum-free medium for 24 hours, the cell monolayer was wounded with a 10 μl pipette tips. Fresh medium was replaced. The wound-closing procedure was observed for 48 hours and photographs were taken at 0 h, 48 h after wounding to determine the wound-closing procedure.

### Transwell assay

Cell invasion ability was detected by the transwell assay (Millipore, MA, USA). Cells were treated with indicated conditions and then seeded on the upper insert coated with 2% Matrigel (BD Biosciences, NY, USA) in 24-well plates (5000/well). The upper insert was filled with medium lacking serum and the lower chamber was filled with 600 μL DMEM supplemented with 10% FBS. After 24 h of incubation, cells invaded to the lower chambers were fixed with methanol, stained with crystal violet.

### Quantitative real-time PCR

Total RNAs were extracted using Trizol Reagent (Invitrogen, CA, USA) according to the manufacturer's instruction. 1 μg total RNAs was reversely transcribed to cDNA using a RNA PCR Kit (Takara, Dalian, China) which was used as a PCR template. To detect gene expression, quantitative real-time PCR (qRT-PCR) was performed using an iCycler iQ System with the iQ SYBR Green Super Mix (BioRad, CA, USA) according to manufacturer's instructions. Small endogenous nuclear U6 snRNA was used as internal control for normalization of miRNA and GAPDH for mRNAs. The relative gene expression levels were calculated using (2^-ΔΔCt^) method.

### Western blot

The protein samples were extracted from the A375 and M14 cells using a lysis buffer. Amounts of 40 μg protein was separated in 10% sodium dodecyl sulfate-polyacrylamide gel electrophoresis and transferred to polyvinylidene fluoride membranes. The blots were incubated with 5% skimmed milk in PBS for 2 h to block non-specific binding followed by probing with primary antibodies (Cell Signaling Technology, USA) overnight at 4°C. After washed with TBST, the blots were incubated at room temperature for 90 min with horseradish peroxidase-conjugated goat anti-rabbit antibodies (Cell Signaling Technology, USA). GAPDH (Cell Signaling Technology, USA) was used as an internal control. Finally, the blot was treated with ECL plus reagent (Pierce, Rockford, IL, USA) and detected using a chemiluminescence detection kit (KPL, Gaithersburg, MD).

### Luciferase assay

The wild-type and mutated 3'UTRs of *STAT3* were subcloned into the pGL3 vector (Promega, WI, USA). Cells were co-transfected with the plasmid constructs of pGL3-*STAT3*-3'UTR or pGL3-*STAT3*-3'UTR-mut and ad-miR-181 or ad-control using Lipofectamine 3000 (Invitrogen, CA, USA). Subsequently, cells were transfected with 0.1 μg PRL-TK (TK-driven Renilla luciferase expression vector) as an internal control. Luciferase activities were measured 48 h after transfection with a dual luciferase reporter assay kit (Promega, WI, USA).

### *In vivo* study

30 Balb/c nude male mice were maintained under a specific pathogen-free condition and randomly divided into three groups. All experiments were approved and carried out according to the guidelines of the Ethics Committee of The Affiliated Hospital of Southwest Medical University. A375 and M14 cells in the amount of 1×10^6^ were subcutaneously injected into the flank region of the mice. IL22 dissolved in normal saline was administered to these mice everyday at the dose of 50 mg/kg for 10 days before tumor inoculation until the end of the study. The mice in IL22+sh-miR-181 group were tail injected with adi-sh-miR-181 after IL22 administration. The tumor sizes were measured every two days using a caliper. The mice were sacrificed at day 30 and tumors were excised. Mice were placed in a Plexiglas chamber with 5% isoflurane (VetOne, Shanghai, China) for 5 min, and decapitated when fully sedated, as measured by a lack of active paw reflex. A part of the issues was placed in 10% formalin for histological and the remaining was frozen in -80 °C.

### Statistical analysis

All statistical analyses were performed with GraphPad Prism (GraphPad software 3.0; San Diego, CA, USA). All the data are presented as the means±SD. One-way ANOVA was used to assess the difference between multiple groups. Differences between two groups were analyzed by the Student's t-test. P<0.05 was considered as statistical significance.

## Results

### Elevated IL22 is detected in both serum of CM patient and CM tissue

**Figure 1:**
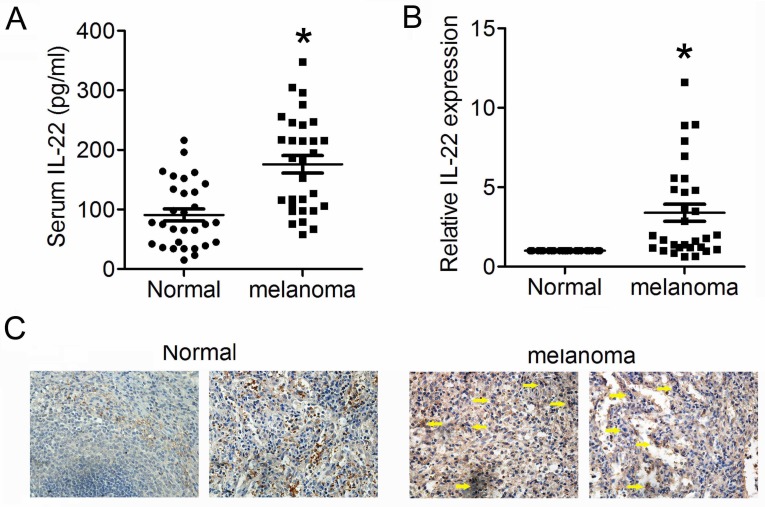
** Elevated IL22 in serum of CM patient and CM tissue.** (A) IL22 expression in serum of CM patients evaluated by ELISA. (B) IL22 mRNA in CM tissues detected by qRT-PCR. (C) IL22 protein expression in CM tissues detected by IHC (×200). *P<0.05 verse normal group.

We first detected expression levels of IL22 in tissues from CM and adjacent normal skin, as well as in serum from CM patients and health control. As shown in Figure 1A and 1B, expression levels of IL22 dramatically elevated in both serum and tissues from CM patients. Consistently, IHC analysis also showed obviously enhanced expression of IL22 in CM tissues (Figure 1C). These results indicate that IL22 is up-regulated in CM.

### miR-181 is up-regulated in the serum of melanoma patient and the melanoma tissue

**Figure 2:**
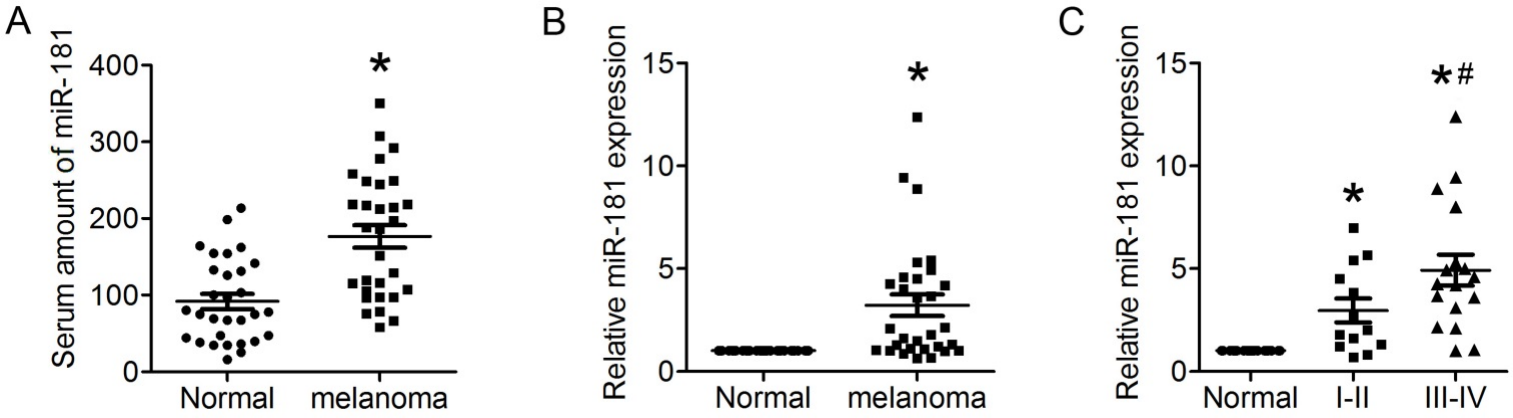
** Up-regulated miR-181 in serum of CM patient and CM tissue.** (A) Expression of miR-181 in serum of CM patients detected by qRT-PCR. (B) Expression of miR-181 in CM tissues detected by qRT-PCR. (C) Expression of miR-181 in stage I-II CM tissues and stage III-IV CM tissues. *P<0.05 verse normal group, ^#^*P*<0.05 verse stage I-II CM group.

IL22 have been identified as an effecter secreted by CD4^+^ T cells that promotes lung tumorigenesis^20^. Recent studies found miR-181 with a clear effect on CD4^+^ T cell development and homeostasis, and involved in cellular apoptosis, differentiation and tumorigenesis^21^, ^22^. To identify whether miR-181 expression is associated with up-regulated expression of IL22 in CM, qRT-PCR was preformed to evaluate the relative mRNA expression of miR-181 in both serum and tissues from CM patients. The present results demonstrated significantly increased levels of miR-181 in both serum (Figure 2A) and tissues (Figure 2B) from CM patients. Moreover, expression levels of miR-181 in tissues from stage III-IV CM were remarkably higher than that from stage I-II CM (Figure 2C).

### IL22 promotes cell proliferation, migration, invasion, and and clonogenic ability in CM cells via up-regulated miR-181

**Figure 3:**
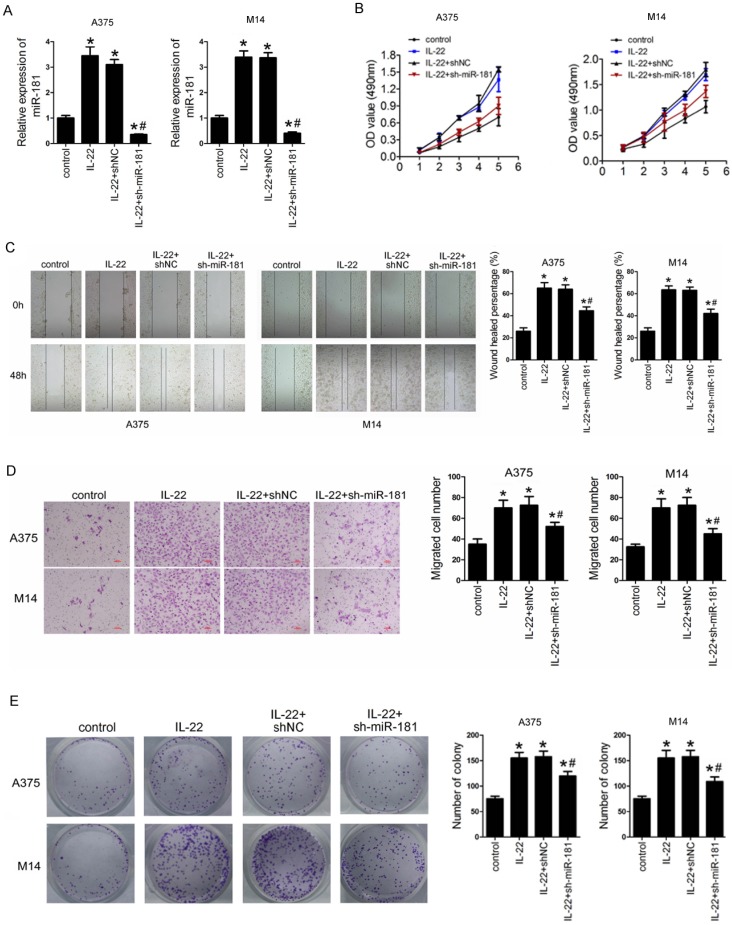
** IL22 promotes cell proliferation, migration, invasion, and clonogenic ability in A375 and M14 cells via up-regulated miR-181.** (A) Expression of miR-181 in A375 and M14 cells under IL22 and ad-sh-miR-181 treatment. (B) Cell proliferation in A375 and M14 cells detected by MTT. (C) Cell migration in A375 and M14 cells detected by wound healing (×100). (D) Cell invasion in A375 and M14 cells detected by transwell assay (×200). (E) Clonogenic ability in A375 and M14 cells. *P<0.05 verse control group, ^#^*P*<0.05 verse IL22+shNC group.

To identify the roles of IL22 and miR-181 in CM progression, we first preformed sh-miR-181 oligonucleotide transfection to establish stable low expression of miR-181 in selected CM cell lines (including A375 and M14) deriving from primary CM^23^ and metastatic CM^24^. As shown in Figure 3A, IL22 treatment notably increased expression of miR-181 in A375 and M14, while sh-miR-181 transfection significantly reduced expression of miR-181. Next, we evaluated the effect of IL22 and miR-181 on cell proliferation, migration, invasion and and clonogenic ability in A375 and M14 cells. The results indicated that IL22 treatment promoted capabilities of cell proliferation (Figure 3B), migration (Figure 3C), invasion (Figure 3D), and clonogenic ability (Figure 3E) in A375 and M14 cells. Reversely, miR-181 downregulation significantly suppressed IL22-induced cell proliferation (Figure 3B), migration (Figure 3C), invasion (Figure 3D), and clonogenic ability (Figure 3E) in A375 and M14 cells. These results identify that IL22 accelerated CM cells progression through up-regulated miR-181.

### IL22-induced miR-181 upregulation targets STAT3/AKT pathway

**Figure 4:**
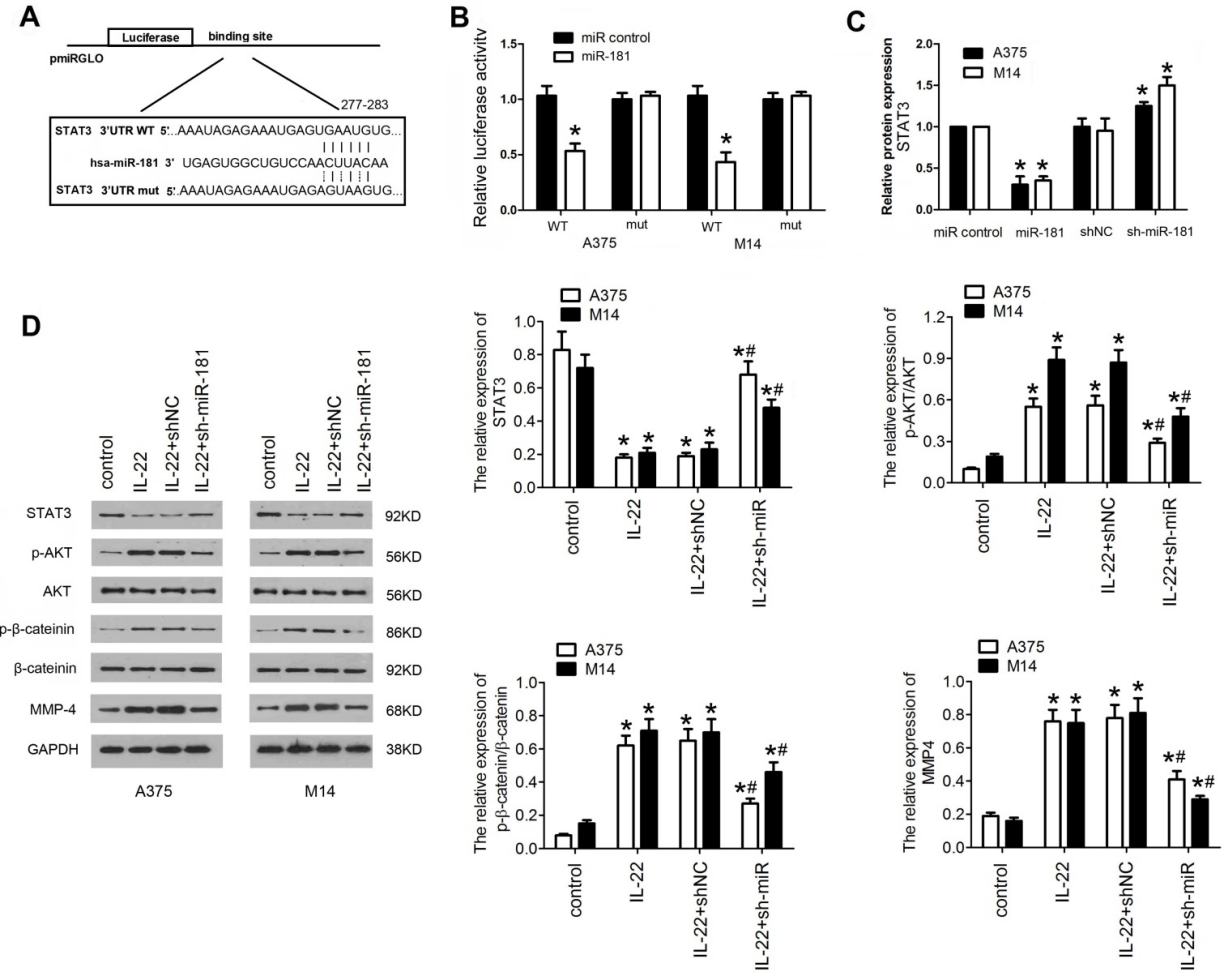
** STAT3/AKT branch is activated in A375 and M14 cells under IL22 teatment.** (A) The target sequence of miR-181 in 3'UTR region of STAT3. (B) Luciferase activities of reporter vectors in A375 and M14 cells. (C) Expression of STAT3 in A375 and M14 cells under ad-sh-miR-181 treatment. (D) Expression of p-AKT, p-β-catenin, and MMP-4 in A375 and M14 cells under IL22 and ad-sh-miR-181 treatment. *P<0.05 verse control group, ^#^P<0.05 verse IL22+shNC group.

To gain insight into the mechanism underlying the effects of IL22-induced miR-181 upregulation on CM progression, potential gene of miR-181 targeted was predicted by bioinformatic algorithms (Targetscan v7.2), and *STAT3* was selected as a target (Figure 4A). To further confirm whether miR-181 directly targets *STAT3*, a firefly luciferase reporter was constructed containing a wild type or mutated type fragment of the 3'-UTR of *STAT3* mRNA. The wild type or mutated type luciferase reporters were co-transfected into A375 and M14 cells with ad-miR-181 or ad-control. The data showed that co-expression of ad-miR-181 with wild type 3'UTR but not with mutant 3'UTR significantly inhibited the luciferase activity in A375 and M14 (Figure 4B). In addition, qRT-PCR was carried out to verify the result; as shown in Figure 4C, STAT3 mRNA expression was remarkably inhibited by miR-181 overexpression and recovered by depletion of miR-181. Since STAT3/Akt signaling axis has been identified as crucial step in cancer development and progression, and been found to be a mediator of IL22-induced effects as well^12^, ^25^, ^26^, we detected the expression of downstream proteins in STAT3 signing including p-AKT, p-β-catenin,. Western blot assay revealed that IL22 treatment significantly inhibited the expression of STAT3, while elevated expression of p-AKT, and matrix metalloproteinase (MMP)-4; however, miR-181 knockdown significantly reversed these effects of IL22 on STAT3/Akt pathway (Figure 4D). These findings indicate that IL22-induced CM progression is regulated, at least partially, by activation of miR-181/STAT3/Akt pathway.

### IL22 promotes CM growth via miR-181 upregulation *in vivo*

**Figure 5:**
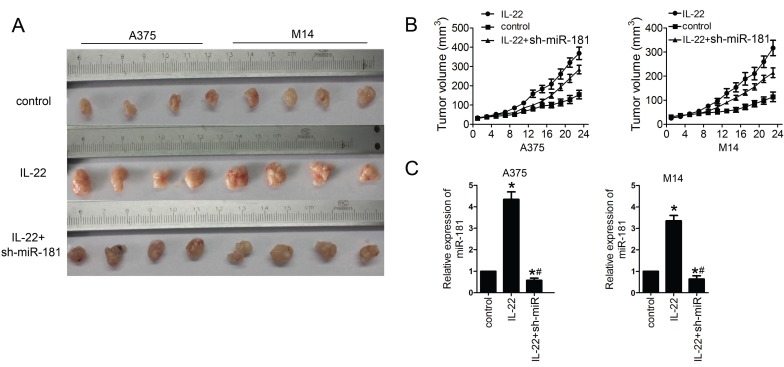
** IL22 promotes tumor growth via miR-181 upregulation in CM xenograft models.** (A, B) Tumor growth in different CM xenograft models under IL22 and ad-sh-miR-181 treatment. (C) Expression of miR-181 in tumor tissues from different CM xenograft models. *P<0.05 verse control group, ^#^*P*<0.05 verse IL22 group.

Finally, we examined the effects of IL22 on CM progression *in vivo*. For this reason, models of CM xenograft in nude mice were developed by subcutaneously injection of A375 and M14 cells. As shown in Figure 5A and 5B, IL22 treatment significantly promoted the tumor growth in CM models; however, sh-miR-181 treatment notably suppressed the effects of IL22 on CM mice. Moreover, qRT-PCR assay results showed that IL22 treatment indeed increased expression of miR-181 in tumor tissues from CM mice (Figure 5 C).

## Discussion

Previously, IL-22 expression was restricted to innate and adaptive immune cells, while the IL-22R seems to be confirmed in non-hematopoietic cells of the skin, lung, kidney and liver^27^, ^28^. Recently study has revealed high expression levels of IL22 in both lung tumors and serum of patients with lung cancer, promoteing *Kras* mutant lung tumorigenesis^20^. Cutaneous melanoma is obviously different from ocular melanoma and mucosal melanoma in epidemiological features, clinical characteristics, treatment and outcomes^29^, ^30^. Although the direct impact and molecular mechanisms of interleukin 22 on human malignancies had been extensively investigated in a great number of previous studies, few report aim at the direct role of interleukin 22 on cutaneous melanoma. To our knowledge, this is the first report show that IL22 promoted cutaneous melanoma cell proliferation, migration, invasion, and increase tumor size *in vivo* as well.

In the present study, we found that expression of IL22 were significantly higher in CM tissue and serum of CM patient. Although IL-22 was frequently observed in both squamous cell carcinomas and basal cell carcinomas, and was confirmed with obvious impacts on them; to our knowledge, this is first report for detailed associations between IL22 and CM. In the present study, we also evaluated the IL22 mRNA expression level from two online TCGA databases (UALCAN cancer database: http://ualcan.path.uab.edu/index.html; and The Human Protein Atlas, www.proteinatlas.org), however, both of these databases have no data about it. Since IL22 was found mainly produced by CD4^+^T cells in tumor-bearing lung tissues, with a key role in modulation of pro-tumor inflammatory responses in lung cancer^20^, our results suggest the probability of IL22 expression being related to dysfunction of tumor-related CD4^+^T cells in CM. Interestingly, as a previous study shown, naive tumor-specific CD4^+^ T cells could differentiate into cytotoxic T cells for melanoma clearance^31^, potentially supporting our opinion. Our data also showed that IL22 treatment promoted cell proliferation, migration, invasion, and colony formation in primary CM cell line A375, and in metastatic CM cell line M14 as well. Consistently, IL22 treatment significantly promoted the tumor growth *in vivo* CM models. These data strongly suggest the suppress effects of IL22 on CM progression.

There are growing evidences that altered expression of miRNAs is strongly linked with carcinogenesis and progression of human malignancies including CM^32^. Additionally, previous researches have linked distinct sets of miRNAs with specific functions of IL-22 in biological processes^33^, ^34^. In the present study, we found up-regulated miR-181 accompanied by significantly increased expression levels of miR-181 in both CM serum and tissues. Moreover, expression levels of miR-181 in stage III-IV CM tissues were remarkably higher than stage I-II CM. Importantly, we showed down-regulated miR-140-3p significantly suppressed IL22-induced cell proliferation, migration, invasion, and colony formation in A375 and M14 cells. Although miR-181 miRNA family was report with clear effects on CD4^+^T cell development and homeostasis, at least partly, similar to the functions as IL-22 shown^35^; our results demonstrate that miR-181 could be employed as a prognostic biomarker for CM development, as well as a predictor for CM outcome.

It has been identified that effect of IL22 on tumor progression is mediated through STAT3 signaling, inducing phosphorylation of kinases AKT and Erk1/2^20^, ^36^. However, the role of IL22 and miR-181 in CM progression is not fully understood. In the current study, we first examined the potential target of miR-181 in CM cell. Both bioinformatics analysis and luciferase reporter assay identified *STAT3* as the target gene of miR-181 in CM cells. Moreover, IL22 treatment significantly inhibited the expression of STAT3, while miR-181 knockdown significantly reversed this effect. Furthermore, IL22 treatment elevated expression of p-AKT, p-β-catenin and MMP-4; however, miR-181 knockdown significantly inhibited phosphorylation of Akt and β-catenin, and expression of MMP-4 as well. Although our results showed IL-22 treatment indeed as an activator of STAT3/Akt signing pathway, interestingly, we noticed that the activation of STAT3/Akt pathway in CM cells was mainly mediated directly by miR-181. Be similarly to this result, other study also indicated an over-expressed miR-181a in CD4^+^T cells could induce phosphorylation of ERK^37^. Previous studies have identified inhibition of β-catenin signaling and matrix metalloproteinase contributing suppressed roles in both migration and invasion of melanoma cells^38^, ^39^. Consistently, we observed increased expression of p-β-catenin and MMP-4 in CM cells under IL22 treatment, revealing the underlying mechanism of IL22-induced CM progression. Interestingly, although many studies have linked diverse subset of MMP to invasion of CM^40-42^, to our knowledge, this is the first report for the role of MMP-4 in CM progression; however, the exact molecular mechanisms implicated in it require additional mechanistic studies.

In summary, our findings provide a new insight into the mechanisms underlying IL22-induced CM progression. Given the potent pro-tumor value of IL22 and miR-181 in CM, them may serve either as prognostic biomarkers or as potential therapeutic strategies for CM patients.
